# Linking Hydrothermal Geochemistry to Organismal Physiology: Physiological Versatility in *Riftia pachyptila* from Sedimented and Basalt-hosted Vents

**DOI:** 10.1371/journal.pone.0021692

**Published:** 2011-07-14

**Authors:** Julie C. Robidart, Annelys Roque, Pengfei Song, Peter R. Girguis

**Affiliations:** 1 University of California Santa Cruz, Department of Ocean Sciences, Santa Cruz, California, United States of America; 2 National Institutes of Health, Vaccine Research Center, Bethesda, Maryland, United States of America; 3 Harvard University, Cambridge, Massachusetts, United States of America; Institute of Marine Research, Norway

## Abstract

Much of what is known regarding *Riftia pachyptila* physiology is based on the wealth of studies of tubeworms living at diffuse flows along the fast-spreading, basalt-hosted East Pacific Rise (EPR). These studies have collectively suggested that *Riftia pachyptila* and its chemoautotrophic symbionts are physiologically specialized, highly productive associations relying on hydrogen sulfide and oxygen to generate energy for carbon fixation, and the symbiont's nitrate reduction to ammonia for energy and biosynthesis. However, *Riftia* also flourish in sediment-hosted vents, which are markedly different in geochemistry than basalt-hosted systems. Here we present data from shipboard physiological studies and global quantitative proteomic analyses of *Riftia pachyptila* trophosome tissue recovered from tubeworms residing in the EPR and the Guaymas basin, a sedimented, hydrothermal vent field. We observed marked differences in symbiont nitrogen metabolism in both the respirometric and proteomic data. The proteomic data further suggest that *Riftia* associations in Guaymas may utilize different sulfur compounds for energy generation, may have an increased capacity for energy storage, and may play a role in degrading exogenous organic carbon. Together these data reveal that *Riftia* symbionts are far more physiologically plastic than previously considered, and that -contrary to previous assertions- *Riftia* do assimilate reduced nitrogen in some habitats. These observations raise new hypotheses regarding adaptations to the geochemical diversity of habitats occupied by *Riftia*, and the degree to which the environment influences symbiont physiology and evolution.

## Introduction

*Riftia pachyptila* is among the best studied of chemoautotrophic symbioses. This siboglonid tubeworm was first described in 1981 [1], [2], and since then has been the subject of numerous investigations (for review see [3]). Briefly, *Riftia pachyptila* (from a monospecific genus hereafter referred to simply as *Riftia*) is the dominant megafaunal species at many sites, growing in enormous aggregations and hosting numerous other species such as mussels, polychaete worms, limpets, and crabs [4]–[6]. Adult *Riftia* are devoid of a mouth or digestive tract, and therefore cannot ingest particulate organic matter [6]. Rather, they possess intracellular chemoautotrophic bacteria within a highly vascularized organ called the trophosome, which ensures effective exchange of metabolites between the symbionts and the circulating hemolymph [1], [2], [7]. The trophosome accounts for between 10 and 30% of the wet tissue weight in *Riftia* depending upon the worm size [7], [8]. Lacking any means of ingesting organic matter, and living where dissolved organics are too low to support the observed growth rates, *Riftia* appears to rely entirely on its symbionts for nutrition. Because the symbionts are not in contact with the external milieu, all their substrates and waste products are provided for or eliminated by the host *Riftia*.

Studies of *Riftia* have focused on their ecology [4]–[6], [9], evolution [10]–[12], and physiology [7], [8], [13]–[19]. Specifically, respirometric, enzymatic and molecular studies have implicated hydrogen sulfide and bisulfide as the reductant, and dissolved inorganic carbon as the source of carbon [13], [14], [16], [18]. Dissimilatory nitrate reduction has been verified as the source of nitrogen -as well as an oxidant- for this association [20]–[24]. These biochemical and physiological studies suggest that *Riftia* is a metabolic specialist, fixing carbon at extremely high rates *via* the Calvin Benson Bassham (CBB) cycle by flourishing in environments where ample hydrogen sulfide, oxygen and nitrate support symbiont chemolithoautotrophy.

Two recent studies have examined the symbionts' population genome (or metagenome, [24]) and proteome [25]. According to the results of the metagenomic analyses, *Riftia's* symbionts' predicted physiological capacity is extremely versatile: genes were recovered that enable the use of organic carbon and thiosulfate, in addition to sulfide, as reductants for energy generation [24]. The proteomic study and associated enzyme activity assays indicate a reliance on the reverse tricarboxylic acid (rTCA) cycle as well as the Calvin-Benson-Bassham (CBB) cycle for carbon fixation [1], [25].

*Riftia* tubeworms are found over an expansive range along ridge systems, from 27°N in the Guaymas Basin to 32°S on the East Pacific Rise [26], [27]. All of the research on *Riftia* physiology and biochemistry to date has focused on individuals found at diffuse vents along the East Pacific Rise, generally between 9 to 13°N, as well as the Galapagos Rift (see above references). The geochemistry of such basalt-hosted hydrothermal diffuse flows have been well-characterized, typically exhibiting higher hydrogen sulfide concentrations, lower concentrations of oxidized sulfur compounds, as well as lower concentrations of dissolved organic carbon and ammonium [28]–[32]. However, *Riftia* are also found in the Guaymas basin at sedimented hydrothermal sites, which have very different geochemistries than basalt-hosted systems. At Guaymas, vent fluids percolate through layers of organic-rich sediment [33], [34], resulting in a geochemically distinct endmember fluid that has a relatively elevated pH, higher organic carbon (recalcitrant and low molecular weight hydrocarbons) and ammonium concentrations, and lower sulfide concentrations in the overlying waters [31], [34]–[37]. At sedimented hydrothermal systems, *Riftia* are often partially buried in sediments (Figure S1), and do not appear to contain comparable amounts of elemental sulfur in their trophosomes when compared to *Riftia* recovered from the East Pacific Rise (*Robidart pers. obs.*).

These distinct geochemical characteristics influence microbial and megafaunal ecology, as evidenced by the differences in the composition and distribution of both microbial and megafaunal species at basalt versus sedimented hydrothermal sites [5], [6], [32], [38]. We further hypothesize that differences in geochemistry would likely lead to differences in microbial and organismal physiology, as the differences in reductants, oxidants and organic carbon might favor different metabolic reactions.

To better understand the degree to which differences in local geochemistry influence *Riftia* chemoautotrophic metabolism (both symbiont and host, with an emphasis on the symbiont), we conducted a series of shipboard high-pressure respirometry experiments during expeditions to 9°N, East Pacific Rise (referred to as EPR throughout this text) and Guaymas Basin. To augment these experimental data, we collected *Riftia* from each site and employed tandem mass spectrometry to characterize and quantify differences in global protein expression of both symbiont and host from freshly-collected specimens. Considering *Riftia's* symbionts' putative metabolic versatility, we posit that their metabolic activity will reflect the local milieu, in particular the availability of organic carbon and reduced nitrogen in the Guaymas Basin. For example, Guaymas sediments are replete in ammonium [31], and the degree to which nitrate is assimilated and utilized by the Guaymas *Riftia* remained unknown prior to this study. Moreover, the symbionts may utilize different forms of sulfur due to more oxidizing conditions around the *Riftia* tubeworm aggregations in Guaymas [31]. This combined experimental and proteomic study sought to determine the differences in symbiont (and host) physiology between these two types of hydrothermal regimes, with a focus on understanding the physiological differences among *Riftia* at the Guaymas Basin (sedimented) and EPR (basalt-hosted) hydrothermal sites.

## Results

### Guaymas and EPR *Riftia* tubeworm site characteristics

During an expedition to the hydrothermal vent sites along the EPR and Guaymas basin in 2008, the *DSV Alvin* was used to collect co-registered micro-scale fluid samples and specimens of *Riftia* tubeworms from among thriving aggregations (*N* = 3).

At the EPR, pH of the seawater from around the *Riftia* plumes was measured at 5.98±0.11, sulfide concentrations averaged 226.11 µM±71, and temperatures were approximately 6°C. At the Guaymas basin, pH of the seawater from around the *Riftia* plumes was measured at 7.12±0.04, sulfide was undetectable, and temperatures were approximately 14°C. Notably, *Riftia* specimens collected from the Guaymas basin were partially buried in the sediment, with a large proportion of their tubes residing beneath the surface while *Riftia* at the EPR were living upon cracks in the basalt (Figure S1b and inset). At the EPR, most *Riftia* were small in size (ca. 15–30 cm in length and 2 cm in width; Figure S1a) and dispersed among *Tevnia jerichonana* tubeworms. These EPR individuals did have the qualitative attributes typical of other *Riftia* collected from this site, including pronounced red plumes, straight thick tubes and elemental-sulfur laden trophosomes (as in [39]).

### Protein quality and proteomic analyses

To ensure that our subsequent analyses were not compromised by poor preservation, we examined protein quality via gel and capillary electrophoresis. These data revealed that protein quality was very high –with very little degradation- due to the rapid dissection and flash freezing of samples (Figure S2). Herein “peptides”, “peptide counts”, and “peptide abundances” refer to peptide-spectrum matches, while the word “proteome” refers to the total identified proteins from the MS-MS spectra that meet our quality criteria (detailed in the methods). To ensure appropriate comparison between Guaymas and EPR *Riftia* symbiont proteins (to avoid mis-estimates in symbiont spectral identification due to differences in host protein abundance in the proteomes), all percentages of symbiont peptides are represented with respect to the total symbiont protein abundance. All interpretations of protein abundances are based on the number of peptide-spectrum matches that identify particular proteins in this study. For a reference database, we used both the previously published *Riftia* symbionts' metagenome [24], as well as a metagenome derived from Guaymas *Riftia* symbionts. Quadruplicate biological and technical replicates allowed verification of results and ensured robust quantification (Figure S3). The ‘housekeeping proteins’, DNA-directed RNA polymerase (alpha and sigma subunits) and the molecular chaperone *dna*K, were normalized to within a standard deviation of 0.1 (0.5±0.4, 0.2±0.7, and 0.3±0.3, respectively), thus supporting usage of the total peptide count ratio for normalization.

### Symbiont nitrogen, sulfur and carbon metabolism

**Figure 1:**
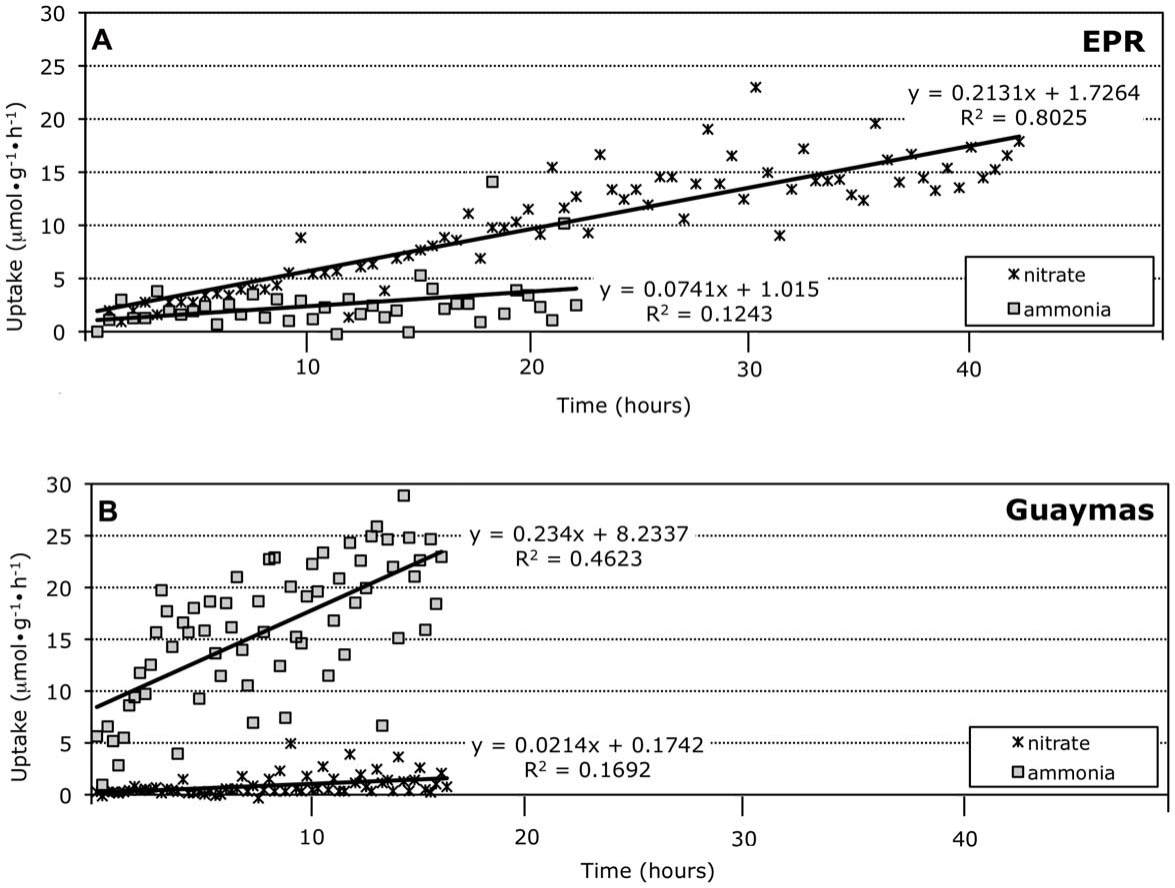
Nitrate and Ammonia uptake by Guaymas and East Pacific Rise (EPR) *Riftia pachyptila*. Tubeworms were collected and quickly placed into high-pressure vessels, and were provided with either ammonium or nitrate (see methods for details). A) Uptake rates observed among EPR *Riftia*, B) uptake rates observed among Guaymas *Riftia*.

Nitrate- and nitrite-reductases, symbiont enzymes involved in nitrogen reduction and assimilation, were 6.9 times more abundant in the symbionts of *Riftia* from EPR than Guaymas basin. Moreover, results of the shipboard incubations demonstrate that EPR *Riftia* acquired both nitrate and very modest amounts of ammonia from the experimental vessel seawater (Figure 1). EPR *Riftia* exhibited a greater uptake of nitrate versus ammonia over the same concentration range, and neither nitrate nor ammonia uptake showed signs of saturation. These data are consistent with previous findings in which net nitrate uptake and minimal ammonia uptake (and typically ammonia production) was observed during shipboard high pressure incubations [23]. In contrast to the *Riftia* from EPR, *Riftia* from Guaymas took up ammonia at a substantially elevated rate, exhibiting a 3-fold greater response to increasing ammonia concentrations, but did not take up nitrate at measurable rates (our limits of resolution for nitrate quantification are ca. 10 nM).

**Figure 2:**
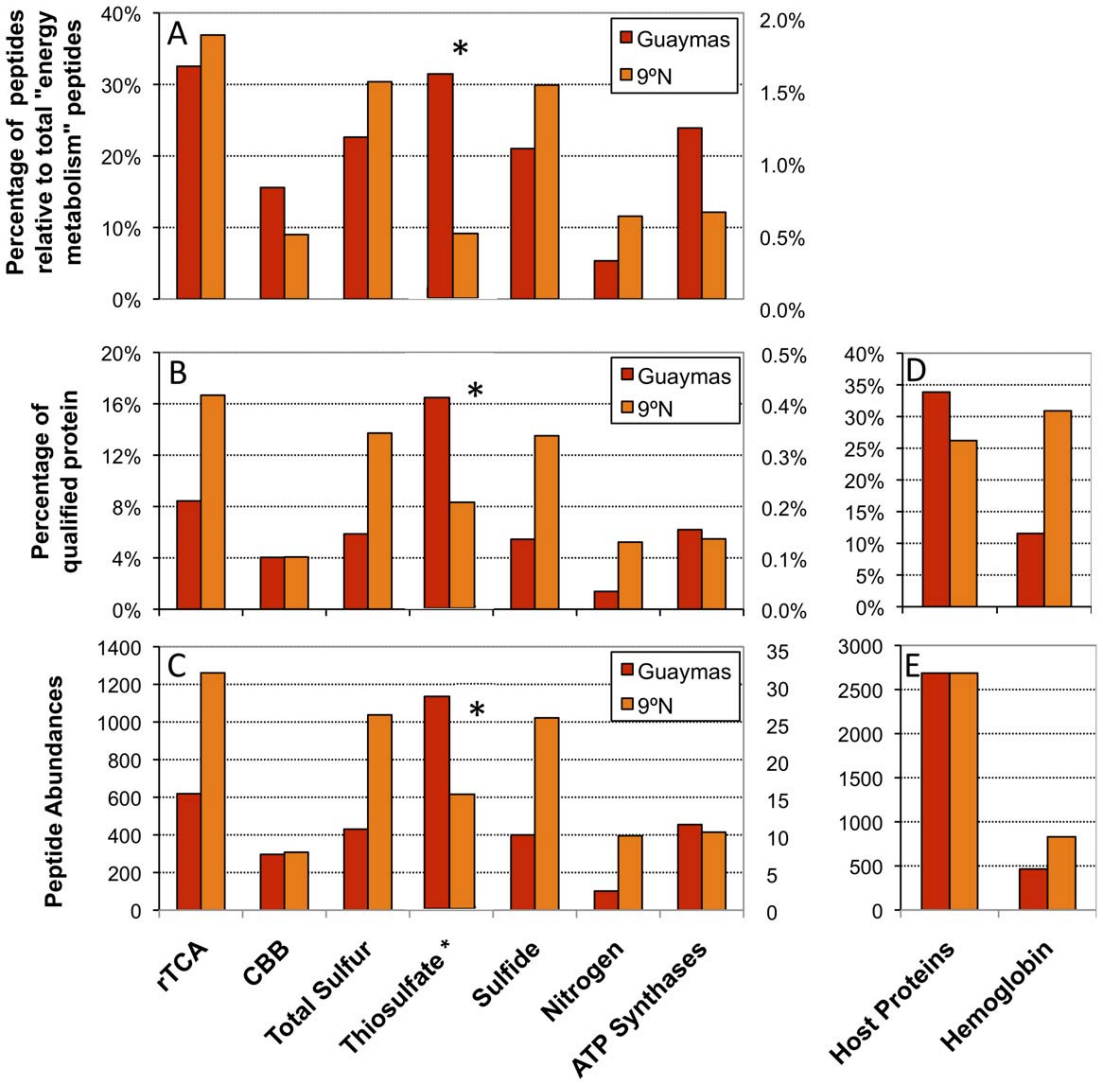
Comparing the Abundance of symbiont and host proteins recovered from Guaymas and East Pacific Rise (EPR) *Riftia pachyptila*. The categories along the x-axes refer to general or specific physiological processes. “rTCA” refers to reductive Tricarboxylic Acid cycle, and “CBB” refers to the Calvin Benson Bassham cycle. “Peptide abundances” refers to the number of peptides allied to qualified proteins associated with each process. “Qualified proteins” refers to those proteins that met the requisite criteria (see methods). Symbiont proteins were evaluated against the total, identified symbiont proteins, host hemoglobin against host proteins, and total host proteins against the sum of both host and symbiont proteins. A) Percentage of peptides, allied with the physiological processes shown on the x-axis, relative to all proteins categorized by C.O.G. as allied to “energy metabolism”, B) Percentage of qualified symbiont protein associated with each category, C) Absolute number of peptides associated with each category, D) Percentage of qualified host protein allied to the host proteome, or specifically hemoglobin, and E) Absolute number of peptides allied to the host proteome, or specifically hemoglobin. Lists of proteins quantified for metabolic categories can be found in Table S1. * = Thiosulfate plotted on secondary axis.

Elemental sulfur abundances in EPR and Guaymas *Riftia* were significantly different. Elemental sulfur abundances in EPR *Riftia* were 4.7±0.9% wet weight, versus 0.7±0.5% wet weight in Guaymas *Riftia* (*N* = 3). Moreover, sulfide and sulfur oxidation enzymes together comprised 8.5% of Guaymas and 13.7% of the EPR symbiont proteome. Peptides highly allied to an enzyme known to be involved in thiosulfate oxidation (*sox* Y; [40]), a well as a Rhodanese transferase found to be involved in dissimilatory thiosulfate metabolism [41] were 2.5 times more abundant (from peptide counts) in Guaymas than EPR symbionts (Figure 2).

Potential rTCA enzymes dominated each proteome, making up 12.2% of the Guaymas and 16.7% of the EPR symbiont proteome. Of these enzymes, those that function in the reductive direction (ATP citrate lyase and 2-oxoglutarate oxidoreductase) were 2.5 times more abundant in the EPR proteome (ATP citrate lyase alone was 4.1 times more abundant). Enzymes potentially mediating carbon fixation *via* the CBB cycle made up approximately 5.8% of the Guaymas and 4.1% of the EPR symbiont proteome, though the CBB diagnostic enzymes RuBisCO and ribulose-5-phosphate epimerase had comparable representation (with 82±9 peptides at Guaymas and 90±8 peptides at EPR).

All peptides discussed above had spectral matches that were precisely allied to proteins from the symbiont metagenome, and not the host mitochondria.

### Global Proteome Comparisons

**Figure 3:**
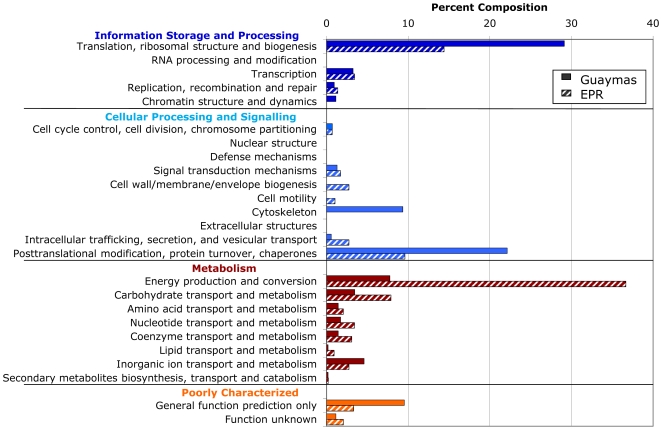
Upregulated proteins recovered from East Pacific Rise (EPR) and Guaymas *Riftia pachyptila* trophosome, categorized using Clusters of Orthologous Groups. Percent composition is with regard to number of occurrences of each protein categorical type, in each proteome. Multiple peptides that were identified as corresponding to a single protein were listed as one hit in the corresponding category.

In order to evaluate the functional roles of the most differentially identified proteins between Guaymas and EPR *Riftia*, proteins were categorized by type using the Clusters of Orthologous Groups database [41] (which was designed for use in analyzing proteins recovered from prokaryotes). The resulting data illustrate the contrasting metabolic poise of the symbionts at each site (i.e. high differential regulation for specific categories; Figure 3). While ‘energy production and conversion’ proteins are prominent in the EPR *Riftia* proteome (comprising 36.6% of the profile), they are noticeably less abundant in the Guaymas *Riftia* proteome (at 7.8%). Proteins involved in ‘transport and metabolism’ of organic compounds (within the organism) in general are more highly represented in EPR *Riftia*. Proteins associated with ‘translation, ribosomal structure and biogenesis’ (mostly ribosomal proteins) are more highly represented in Guaymas *Riftia*. Proteins associated with ‘posttranslational modification, protein turnover, chaperones,’ are also more highly represented in Guaymas *Riftia* (22% in Guaymas *Riftia* vs 9.6% in EPR *Riftia*).

**Figure 4:**
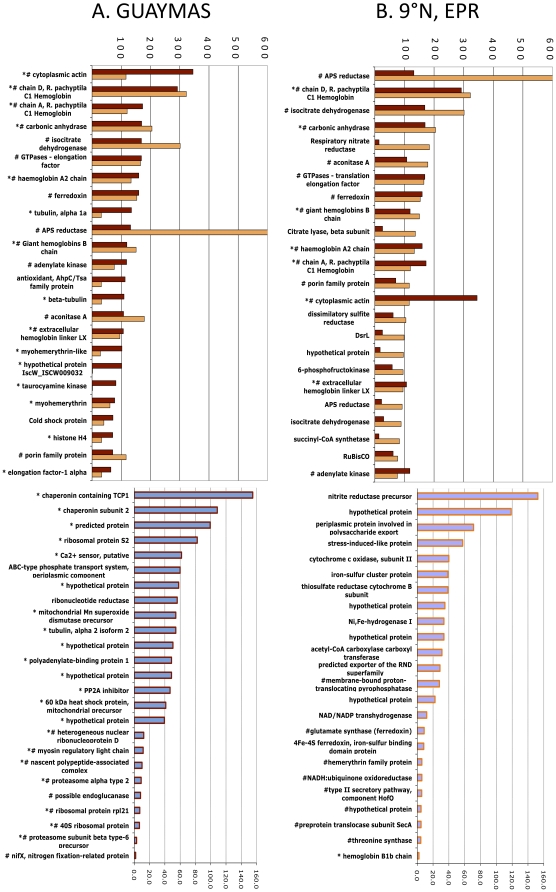
Changes in representation of proteins from East Pacific Rise (EPR) and Guaymas *Riftia pachyptila* trophosome. Bar chart of 25 most abundant peptides organized by A) those most abundant in Guaymas tubeworms and B) those most abundant in EPR tubeworms. Pound signs denote proteins that were identified in both Guaymas and EPR *Riftia* symbiont proteomes, while asterisks denote those proteins that came solely from the host proteomes.

Peptides from each proteome were also independently ranked in order of absolute peptide abundance (Figure 4). Fourteen of the top twenty-five most abundant proteins are shared between both proteomes. These proteins include host hemoglobin and carbonic anhydrase, as well as symbiont APS reductase, aconitase and isocitrate dehydrogenase. Seven of the most abundant proteins recovered from EPR *Riftia*, and sixteen of the top twenty-five recovered from Guaymas *Riftia*, are of host origin.

## Discussion

The data presented here offer a new perspective on the physiological poise of *Riftia* symbioses living in two distinct geochemical settings: basalt- and sediment-hosted vents. These data demonstrate that nitrogen metabolism by *Riftia*, specifically the use of nitrate or ammonia by *Riftia* symbionts, is clearly different between the two sites. Moreover, differences in the representation of proteins associated with carbon fixation and sulfur oxidation pathways further suggest differences in the physiological poise of the symbionts between these two sites. While *Riftia's* symbionts reside deep within the hosts, and *in hospite* are never in direct contact with the environment, these data do suggest that the geochemical milieu does influence symbiont physiology despite the host's pronounced capacity to buffer environmental changes in substrate concentration [7], [16], [18], [42], [43]. In the sections below, we further consider the implications of the observed differences to symbiont nitrogen, sulfur and carbon metabolism.

### Nitrogen metabolism

Prior to this study, substantial ammonia assimilation has never been observed in *Riftia* found at the East Pacific Rise, though glutamine synthetase activity has been demonstrated from plume tissues [44]. Ammonium concentrations are high at sedimented Guaymas vents (∼10–16 mM: [31]) but are very low (≤11 uM) at the basalt-hosted EPR vents [45], and the symbionts' physiologies strongly reflect the geochemistry of their environments (Figure 1). During our respirometric experiments, wherein the individuals were maintained under pressure in hydrothermal conditions until they reached steady state (indicated by a stable sulfide oxidation rate), we observed differential acquisition of nitrate or ammonia by EPR and Guaymas *Riftia*. Nitrate was readily taken up by EPR *Riftia*, while ammonium assimilation was minimal. Guaymas worms exhibited the opposite pattern, acquiring ammonium but not nitrate. In general, ammonia is the preferred substrate for nitrogen assimilation by many organisms -including prokaryotes- because it can be incorporated directly into amino acids *via* glutamine synthetase. Also, animals are incapable of using nitrate or nitrite as a nitrogen source for growth [20]. Nitrate reduction, however, is mediated by bacteria (the symbionts in this case), which can reduce this oxidant to ammonia for assimilation by themselves and their *Riftia* hosts (as in [23]). Nitrate reductase peptides are vastly more abundant in the EPR *Riftia* symbiont proteome relative to Guaymas, suggestive of an increased dependence on nitrate as a nitrogen source in an environment where ammonia is scarce. Specifically, respiratory and assimilatory nitrate reductase peptides are 8.7 and 23.9 times more abundant in the EPR *Riftia* symbiont proteome respectively. Both respiratory and assimilatory nitrate reductases have been implicated in nitrate reduction, and ultimately the production of ammonia [21], [23]. Finally, in light of the observed nitrate and ammonia assimilation rates, as well as the representation of key proteins in the proteome, *Riftia* nitrate or ammonia acquisition may have implications for the chemical milieu around the tubeworm aggregations. At Guaymas, an active, growing clump of *Riftia* living in the slower flowing sediment hosted vents may effectively reduce the concentrations of ammonia in the surrounding seawater (though the degree to which this occurs is unknown). At the EPR, however, production of ammonia from nitrate reduction by this symbiosis contributes to a constant leakage of ammonium into the environment, which may act as a source of reduced nitrogen for free-living organisms [23]. Here again the extent of this process remains unknown.

### Sulfur oxidation

Previous research on *Riftia* found at the EPR suggest that these tubeworms are strictly hydrogen sulfide oxidizers, as the investigators did not observe significant differences in uptake of thiosulfate by *Riftia* (or other reduced substrates such as methane; [13], [14], [39], [46]). Investigators also noted that EPR *Riftia* aggregations flourish where hydrogen sulfide concentrations in the hundreds of micromolar [28], [30], [47]. As mentioned previously, sulfide concentrations around the plumes of EPR *Riftia* were approximately 220 µM while sulfide concentrations were undetectable around *Riftia* plumes at Guaymas. The availability of hydrogen sulfide at the plumes of *Riftia* at the EPR likely translates to a greater supply of hydrogen sulfide to the symbiont. Thiosulfate, a more oxidized sulfur-containing compound, is found in higher concentrations in the overlying waters surrounding Guaymas *Riftia*. Guaymas *Riftia* symbiont enzymes likely involved in thiosulfate oxidation, including the thiosulfate binding protein *sox*Y, are 3.5 times as abundant in Guaymas versus EPR *Riftia* symbionts. As is true for all proteomic analyses, we cannot make inferences about specific activities or flux from protein abundance. Nevertheless, the increased representation of enzymes involved with thiosulfate oxidation suggests that Guaymas *Riftia* may rely on thiosulfate oxidation for energy generation, or may -when appropriate- supplement sulfide oxidation with thiosulfate oxidation for energy production, as has been observed in *Allochromatium vinosum* [48]. Alternatively, *sox*Y has been found to bind sulfur intermediates involved in sulfide oxidation [49], and it may be that this mechanism is transcriptionally controlled: i.e. that the expression of this sulfur-binding protein is increased in response to sulfur starvation. Nevertheless, we suggest that Guymas *Riftia* may be capable of using thiosulfate as a reductant, given the limited availability of sulfide at the plume, and the limited capacity of *Riftia* to take up sulfide at sufficient rates across its body wall (which has much lower surface area than the plume). Notably, the aforementioned thiosulfate stimulation experiments (e.g. 14]) were conducted on *Riftia* collected from high sulfide sites, and these individuals likely had higher amounts of elemental sulfur in the trophosomes as well. These EPR *Riftia* were acclimated to high sulfide conditions, which may have precluded the use of thiosulfate by the association. Future studies of intact Guaymas *Riftia* in high pressure respirometry systems may provide more information on the uptake of sulfide and other sulfur containing compounds.

### Carbon fixation

The results of our study confirm that the key enzymes that mediate both rTCA and CBB are expressed by both Guaymas and EPR *Riftia* symbionts (Figure 2). When comparing the corresponding enzyme abundances between the EPR symbionts in this study (Table S1) and the previously-published study of the EPR *Riftia* proteome [25], marked differences emerge. For example, in the current study of EPR *Riftia* symbionts, approximately 80% of all proteins potentially involved with carbon fixation (both CBB and rTCA pathways) were associated with the rTCA cycle (Figure 2, Table S1). In the previous proteome study, approximately 66% of these potential carbon fixation proteins from *Riftia* symbionts were allied with the rTCA cycle. In this study, the aforementioned CBB and rTCA diagnostic enzymes comprise 20.2% vs. 79.8% of the total carbon fixation proteins respectively (Table S1). In the previous Markert *et al.*, study [25], CBB and rTCA diagnostic enzymes comprise 42.5% and 57.5% of the total carbon fixation proteins respectively. We also observed that Guaymas and EPR *Riftia* symbionts had comparable representation of enzymes involved in CBB, ca. 4.0% (Figure 2), though the enzyme that typically governs rate of carbon fixation *via* CCB, ribulose bisphosphatase carboxylase/oxygenase, or RuBisCO, is about 25% more abundant in EPR than Guaymas *Riftia* symbionts. With respect to rTCA, 16.7% of EPR *Riftia* symbionts versus 8.4% of the Guaymas *Riftia* symbionts' total proteins are potentially involved in the rTCA cycle (Figure 2). ATP citrate lyase and 2-oxoglutarate oxidoreductase both play a key role in the directionality of the TCA cycle to fix inorganic carbon, and these enzymes are respectively 341% and 40% more abundant in EPR *Riftia* than Guaymas *Riftia*. In comparison with the proportion of CBB and rTCA proteins in EPR *Riftia* symbionts (at 20.2% and 79.8% respectively), the Guaymas *Riftia* symbiont CBB diagnostic peptide abundances amount to 38.7% of the total carbon fixation key enzyme abundance, vs. 61.3% for the rTCA (Table S1).

In the previous study, Markert *et al* interpreted the proteomic data and the measured activity of rTCA cycle enzymes to suggest that rTCA and CBB cycles are concurrently active. Previous studies [1] have demonstrated that RuBisCO is also expressed and active in these tissues. While we did not measure any enzyme activities, and thus cannot comment on the activity of key enzymes in CBB or rTCA, the differences in representation of key enzymes between the EPR and Guaymas *Riftia* suggest that these two modes of carbon fixation may be differentially utilized in these environments. This is based on the assumption that differential expression of a protein by similar organisms in different environments may reflect differences in metabolic activity. However, it is known that protein expression is influenced by many factors, and does not necessarily influence flux, so this supposition remains to be tested.

As a matter of consideration, there are two environmental factors that directly bear on the value of having two different modes of carbon fixation. First, there is the question of energetics, in particular whether the availability of reductant affects the use of rTCA or CBB for carbon fixation. The rTCA cycle may have the advantage of a greater number of moles fixed carbon per ATP than the CBB cycle [50], though no study has yet extrapolated that information into comprehensive energetic costs and benefits to the cell. Assuming that it is the case, and given the lower representation of sulfur oxidation proteins in Guaymas *Riftia* symbionts, and the undetectable concentrations of sulfide around *Riftia* plumes, one could speculate that Guaymas *Riftia's* symbionts may be relatively limited in reductants when compared to the EPR symbionts. If sulfide oxidation rates are low and energy limiting, the energetically favorable reverse TCA cycle might have an advantage for Guaymas *Riftia* symbionts, provided that it can function at *in hospite* oxygen concentrations. This is, however, inconsistent with our data, which exhibit the opposite pattern of representation in EPR and Guaymas *Riftia*. The second issue is that of oxygen concentration, in particular the partial pressure of oxygen surrounding the symbionts. In previous studies, it was observed that rTCA enzymes exhibit higher sensitivity to oxygen when compared to RuBisCO [51], [52]. Guaymas *Riftia*, whose plumes are exposed to well oxygenated waters relative to tubeworms at EPR, may have higher partial pressures of oxygen in their tissues that may select for the use of the CBB for carbon fixation to mitigate the effects of higher oxygen by using a less oxygen-sensitive carbon fixation pathway. However, in light of the extremely high affinity for oxygen exhibited by *Riftia* hemoglobins, the partial pressure of oxygen within the tissues may be diminishingly low. Though the threshold for oxygen toxicity may be known for characterized systems, we did not measure the oxygen concentrations within intact *Riftia* trophosomes, nor has any previous investigator. Thus, this hypothesis is beyond the scope of these data to address. Future investigations should aim to use other approaches, e.g. isotopically labeled substrates, to determine the relative contribution of each of these carbon fixation pathways to the tremendous rates of carbon fixation observed in *Riftia*.

Though enzymes in the CBB cycle and the rTCA cycle are represented in the symbiont proteome, phosphoenolpyruvate (PEP) carboxykinase is only present in the host proteome. It is possible that this contributes to condensation of CO_2_ and PEP in the host fluids, much like the mesophyll of plants [15]. Small organic acids can be supplied to the symbiont through TRAP transporters, which are among the abundant qualified proteins in both proteomes. These compounds can enter the reverse TCA cycle anapleurotically, thereby contributing to carbon fixation by the symbiont, as hypothesized previously [24].

### Host energy storage

Phosphagen kinases catalyze the reversible transfer of the gamma phosphoryl group of ATP to naturally occurring guanidino compounds, such as creatine, glycocyamine, and taurocyamine among others, yielding ADP and a phosphorylated guanidine commonly called a phosphagen. High activities of this phosphagen kinase point to enhanced capacity for ATP storage [53]. In the current study, taurocyamine kinase was much more abundant in the Guaymas *Riftia* symbionts proteome (Figure 4), suggesting that ATP is available in excess for “storage” (Figure 2). Alternatively, the low representation of taurocyamine kinase in the EPR *Riftia* symbionts (2 peptides identified in EPR vs. 123 in Guaymas proteome, Figure 4) may be related to the host's need to expend energy on other processes. For example, EPR *Riftia* likely expend far more energy eliminating hydrogen ions than Guaymas *Riftia*. Hydrogen ions are an end product of sulfide oxidation, and must be eliminated to prevent metabolic acidosis. Indeed, hydrogen ion elimination rates by EPR *Riftia* are among the highest ever measured [17], [54]. At the EPR, a significant fraction of *Riftia's* ATP are expended on hydrogen ion elimination, since *Riftia* has to “pump” these out against a concentration gradient (note that the pH of *Riftia* tissues and blood is around 7.4 [16], while the seawater surrounding the plumes is typically more acidic, circa 5.9). At Guaymas, the seawater surrounding *Riftia's* plumes is circumneutral, likely requiring less energy of Guaymas *Riftia* to eliminate hydrogen ions resulting from sulfide oxidation. Proteins represented in our Guaymas and EPR proteome are consistent with this model, as a proton-translocating pyrophosphatase is the most abundant protein recovered in the EPR *Riftia* host proteome (Figure 4). Comparably, only four such peptides were found in the Guaymas *Riftia* host proteins. This supposition should be further tested, but in light of this and previous studies, the model presented is a very likely explanation for the observed differences in proton-translocating pyrophosphatases between Guaymas and EPR *Riftia*.

### Transport

To date, the symbionts are the only known source of organic carbon for their invertebrate hosts, but the transporter families involved in organic carbon translocation from symbiont to host are poorly understood. The transporters most represented in the EPR *Riftia* symbiont proteome include a Resistance, Nodulation and cell Division (RND) superfamily exporter (drug and heavy metal efflux: [55]) a polysaccharide export protein, and a Type II Secretion System (TTSS: involved in pathogenicity in some characterized systems: [56]). This RND exporter type is homologous to a multidrug export type from *Acinetobacter*, though the function of this specific protein in the EPR *Riftia* symbiont is unknown. A second RND superfamily exporter is present in both Guaymas and EPR proteomes, and this version shows the highest sequence similarity to one found in other lithotrophs (as revealed by BLASTS to Genbank non-redundant database) perhaps implying an environmental role such as metal transport. Aside from the polysaccharide export protein, no clear secretion mechanisms were identified for these symbioses. Previous studies using autoradiographic approaches have pointed to carbon release by symbionts immediately after fixation, in addition to bacteriocyte degradation at the peripheries of the trophosome lobules [57]. The lack of identified secretion/transport proteins suggests that they are perhaps among the ‘hypothetical proteins’ in this study, or that carbon is simply leaked through membranes from the symbionts into the bacteriocytes.

### Other aspects of the proteomes and emerging hypotheses

Over half of the 25 most abundant proteins, from host and symbiont, are shared between both Guaymas and EPR proteomes, suggesting their role in key physiological functions (Figure 4). Many of these shared proteins belong to the host and are well-studied proteins (hemoglobin and carbonic anhydrase) whose expression has direct implications for symbiont chemolithoautotrophy *via* sulfide and oxygen transport and carbon dioxide uptake [43], [46], [58]–[61].

While it is well established that *Riftia* are primarily dependent on their symbionts for their nutrition (e.g. [57], [62]), the data presented here demonstrate that at Guaymas, *Riftia* acquire organic nitrogen (ammonium) to meet their needs for biosynthesis. Notably, these data also provide circumstantial evidence that *Riftia* may be acquiring organic carbon from the environment. Specifically, a glycoside hydrolase family 16 protein (commonly known as a cellulase and with highest sequence identity to accession number YP_002018773) was found to be among the most abundant differentially expressed enzymes in the Guaymas *Riftia* proteome (Figure 4). There is a highly conserved cellulose binding domain in this protein, which is suggestive that this enzyme could interact with cellulose. The gene coding for this protein is also present in the same contig as a pectate lyase, highly implicating its identification as a cellulase (though pectate lyase was not identified in the proteome). To determine if Guaymas *Riftia* tissues showed signs of cellulase activity, assays for endo-ß-1,4-glucanase activity with carboxymethylcellulose were run on Guaymas and EPR trophosome, blood and gill (as a control). Cellulase activity was detected within all three trophosomes tested, and appeared to be higher in the Guaymas worms (relative to the EPR worms). However, these activities were highly variable, and not statistically significant relative to the control (namely cellulose activity in the plume, which does not host symbionts; data not shown). Note that cellulases have been shown to have specificity for particular substrates in better characterized systems [63]; future efforts will aim to examine endoglucanase activity using other compounds, e.g. lichenin and lamarin, as substrates.

It is known that Guaymas hydrothermal sediments are rich in total organic carbon [37], [64], [65], and there is no *a priori* reason to believe that organic carbon acquisition is implausible (though prior studies of EPR found no evidence for organic carbon uptake, [13], [14]). While the data shown here are insufficient to state that the observed protein is involved in cellulose degradation, its representation in this proteome, and the nature of the Guaymas vents, begs the question as to its role in this association. While cellulose is abundant in Guaymas sediments, it is both highly stable and insoluble. It may, however, be reduced and made water soluble at hydrothermal conditions similar to those at Guaymas Basin (220–250 MPa, ≥260°C: [66], [67]). Notably, if cellulose or a derivative is available in their environment, it would have to pass through the host before being delivered to the symbiont. Cellulose cannot be metabolized by metazoans and, though the *Endoriftia persephone* genome encodes for all the enzymes necessary for this process [24], dissolved cellulose transport by host cells has not been demonstrated to our knowledge. While this is highly speculative, we present this observation as a suggestion that organic carbon assimilation by sediment-hosted tubeworms is worth investigation in future experiments. Should this be proven true, it would expand our view of chemosymbioses, and provide compelling hypotheses for physiologies of various other chemosynthetic organisms from sedimented habitats.

### Conclusion

Though *Riftia* associations along the East Pacific Rise (EPR) have been extensively characterized (for reviews, see [3] and [68]), and previous data suggest that the host buffers the symbiont from the environment [7], [16], [18], [42], [43], the data in this study demonstrate that local geochemistry necessarily influences symbiont physiology. Moreover, to date there are no comparable studies of *Riftia* found in the Guaymas basin. The trophosome proteome from the Guaymas *Riftia* highlights the paucity of information on the symbiosis at this locality, and raises some significant physiological questions. The differences in the proteomes reflect the physiological activity of each symbiosis in its respective environment, and suggest that the Guaymas *Riftia* symbionts may be less specialized for sulfide than previously thought. More significantly, these data reshape our thinking about the nutritive coupling between host and symbiont, and illustrate that it may be altered by the geochemical milieu. The availability of reduced nitrogen to the Guaymas *Riftia* led to a near cessation of nitrate acquisition, and the presence of an endoglucanase -albeit uncharacterized- raises the question as to whether these associations can and do acquire exogenous carbon. While genomics and proteomics continue to be powerful tools for interrogating a diversity of organisms, including those that are not cultured in the laboratory, the combined use of experimental approaches such as high-pressure incubations at environmentally relevant conditions along with genomic/proteomic analyses provides a highly effective means of testing and generating new hypotheses. To better understand the observed differences in sulfur and carbon metabolism, future studies will couple proteomics and high pressure respirometry to evaluate physiological mechanisms of this symbiosis while determining the biogeochemical outcome of the organisms' metabolic state. Such approaches will help us better understand how these chemosynthetic associations respond to natural variations and perturbations in their environment.

## Methods

*Riftia pachyptila* were collected from 2001 m deep in the Guaymas basin (27°N, 111°24′W) and from 2506 m at the Tica vent along the East Pacific Rise (9°50′N, 104°18′W) during expeditions in October 2007. Worms were sampled by *DSV Alvin* at the end of each dive, and recovered in a well-insulated sampling box, which kept the animals in cold seawater during ascent. *Riftia* tubeworms from Guaymas were collected from a heavily sedimented area, and those from EPR were collected from basalt-hosted diffuse flows (Figure S1). Micro-scale fluid samples were taken with the SIPPER sampler [68] at these sites prior to tubeworm collection, and were analyzed for sulfide and pH using a colorimetric assay [69] and a conventional shipboard pH meter (provided by Susana Serrano and PIs of Extreme 2007). On deck, the temperature within each biobox was recorded and the animals were immediately transferred to ice cold seawater. Worms used in this study were kept below 10°C during recovery and when on deck. Within 5 minutes each tubeworm has been removed from its tube then flash-frozen in sterile packaging at −80°C. Samples were then transported to the lab on dry ice.

Samples were dissected using sterile tools, taking care to avoid thawing. The interior of the trophosome was excised to prevent contamination from the body wall. The trophosome from three Guaymas and EPR worms were individually diluted in ten parts Laemlli buffer (Bio-Rad, Hercules, CA) and heated to 95°C for 10 minutes. The resulting extracts were run on a 15% Tris-glycine gel (Bio-Rad, Hercules, CA). Gels were stained with Colloidal Blue gel stain (Invitrogen, Carlsbad, CA) and bands were excised according to their relative positions in the gel. The resulting excised gel sections were rinsed in ultra high purity acetonitrile, placed into sterile 1.2 mL eppendorf tubes, and stored at −80°C for no more than four weeks prior to submission for analyses by the Harvard Mass Spectrometry and Proteomics Resource Laboratory.

At the proteomics facility, samples were reduced, carboxyamidomethylated and digested with trypsin. Resulting peptides from each sample were analyzed over four technical and biological replicates using microcapillary reverse-phase HPLC directly coupled to the nano-electrospray ionization source of a ThermoFisher LTQ-Orbitrap XL mass spectrometer (μLC/MS/MS). The Orbitrap repetitively surveyed from m/z 395 to 1600, while data-dependent MS/MS spectra on the twenty most abundant ions in each survey scan were acquired in the linear ion trap. MS/MS spectra were acquired with relative collision energy of 30%, 2.5-Da isolation width, and recurring ions dynamically excluded for 60 s. Preliminary evaluation of peptide-spectrum matches (PSMs) was facilitated with the SEQUEST algorithm with a 30 ppm mass tolerance against the NCBI nonredundant protein database (December 2008) and a provided database, constructed specifically for these analyses. This database contains expressed sequence tags (ESTs) from *Ridgeia piscesae* [70], and amino acid sequences from the metagenome of *Endoriftia persephone* (the symbiont) from 9°N, East Pacific Rise [24], and the recently completed metagenome of Guaymas Basin *Riftia* symbionts. In addition, we also analyzed these spectra against the genomes of physiologically- or phylogenetically-affiliated proteobacteria (*Buchnera aphidicola* str. APS (accession number NC_002252), *Methylococcus capsulatus* str. Bath (accession number NC_002977), *Wolbachia* endosymbiont of *Drosophila melanogaster* (accession number NC_002978), *Thiobacillus denitrificans* ATCC 25259 (accession number NC_007404), *Nitrosococcus oceani* ATCC 19707 (accession number NC_007483), *Thiomicrospira crunogena* XCL-2 (accession number NC_007520), *Hahella chejuensis* KCTC 2396 (accession number NC_007645), *Magnetococcus* sp. MC-1 (accession number NC_008576), Candidatus *Ruthia magnifica* str. Cm (accession number NC_008610), Candidatus *Vesicomyosocius okutanii* HA (accession number NC_009465), *Chlorobium limicola* DSM 245 (accession number NC_010803) and *Allochromatium vinosum* (accession number NC_013851)).

With a custom version of the Harvard Proteomics Browser Suite (ThermoFisher Scientific, San Jose CA), protein-spectrum matches (PSMs) were accepted with mass error <3.0 ppm and score thresholds to attain an estimated false discovery rate of ∼1% using a reverse decoy database strategy. Protein sequences from NCBI nr that matched the trophosome spectral output were combined with the matches from our constructed database to create a master database with the collective matches. This master database was then compared with the trophosome spectra in order to create a competitive comparison between the best matches. Peptides were enumerated by spectral counting, and only those meeting the following criteria were considered for subsequent analyses: 1) they must have been identified in all four replicates, and 2) a minimum of three unique peptides must associate to a single protein identification. Peptide counts whose values were outside two standard deviations of the mean were considered outliers and were removed from subsequent analyses. Peptide counts were normalized using the ratio of total peptide counts from each site (e.g. EPR/Guaymas, which was 1.50). To aid in the comparative quantification and analyses, all zero values were exchanged for a threshold value of 0.2. Peptide counts were normalized to a log base 2 in order to more easily determine differential representation of proteins within each sample. To avoid the overestimation of the EPR symbiont protein numbers due to the greater number of host proteins in the Guaymas sample, when addressing differential spectral counts between symbionts these numbers are calculated at the exclusion of host proteins. Screening for quality control (see methods) resulted in the selection of 493 of the identified 1513 proteins for analyses.

For protein functional diversity comparisons (Figure 3), proteins were ranked in order of magnitude of differential expression, and then BLAST was used to compare those proteins upregulated in Guaymas, then those upregulated in EPR, to the Clusters of Orthologous Groups database [71].

### Shipboard high-pressure respirometry experiments

During expeditions to the EPR (9°50′N, 104°18′W) and Guaymas basin (27°N, 111°24′W) sites in 1998 and 2003, *Riftia* tubeworms were collected with *DSV* Alvin from a depth of approximately 2500 and 1900 meters respectively, and recovered as previously described. Worms were immediately placed into flow-through, high pressure respirometer aquaria [18]. All experiments were conducted at 12°C and 27.5 Mpa. One to three tubeworms weighing between 6 and 18 grams each were placed into two of the aquaria of the high pressure respirometry system. Worms were maintained for twenty two to twenty four hours at “*in situ*” vent conditions (∑CO_2_ = 5.5 mM, ∑H_2_S = 240 µM, O_2_ = 135 µM, pH = 6.1 and 6.8 for EPR and Guaymas worms respectively; “∑” is used to indicate the total concentrations of all ionic species of inorganic carbon and sulfide) without any available nitrogenous substrates (other than nitrogen gas). A third vessel, which served as a control, was devoid of tubeworms.

### Measuring rates of nitrate and ammonia uptake by *Riftia*

After acclimation to the pressure vessels, either nitrate or ammonia was added to the effluent to achieve seawater concentrations up to 45 µM, and changes in seawater nitrate and ammonia concentrations were determined during the course of all experiments (which lasted 6 to 14 hours). Sodium nitrate salts were delivered into the equilibration column (as described in [23]), at rates necessary to achieve the desired seawater concentration. Ammonium sulfate salts were delivered to the equilibration column in the same manner, again at rates sufficient to achieve the desired seawater concentration. During and after the experiments, nitrate was quantified by spectrophotometric analysis of discrete water samples [72] collected prior to and after exposure to *Riftia*. Seawater ammonium concentrations were determined by flow injection analysis of discrete water samples [73].

At the end of each experiment, worms were removed from the aquaria, quickly separated from their tubes, and weighed on the motion-compensated balance [74]. All rates and other calculations are expressed in terms of wet weight. Tubeworms were then dissected, and tissue samples were promptly frozen in liquid nitrogen for later analyses. In most cases, the empty worm tubes were returned to the pressure vessel, and subjected to the same experimental conditions to determine what fraction, if any, of our observed flux rates are attributable to bacterial growth or other phenomena associated with the tubes.

### Elemental sulfur quantification

Elemental sulfur in the trophosome of Guaymas and EPR *Riftia* were quantified via gas chromatography as described in [46]. Briefly, frozen Guaymas and EPR *Riftia* trophosomes (three individuals from each site) were dried for 18 h at 100°C, then elemental sulfur was extracted with cyclohexane, cleaned via passage through a fluorosil column and the resulting eluent was injected into a gas chromatograph modified to accept fluid samples [7].

## Supporting Information

Figure S1 ***Riftia*** **tubeworms observed at the East Pacific Rise (EPR) and the Guaymas basin.** **A**) *Riftia pachyptila* observed on the basalt hosted vents at the EPR, B) *Riftia pachyptila* as observed at the sediment hosted vents in the Guaymas basin, 1b) inset show tubeworm buried in sediment to its plume. Images were taken during an expedition to the EPR and Guaymas in 2007. Figure 1b is from an expedition to the Guaymas basin in 2003 (courtesy of MBARI).
(TIF)

Figure S2 **15% SDS-PAGE gel of proteins recovered from Guaymas and East Pacific Rise (EPR)** ***Riftia pachpytila*** **trophosome.** Marker shown is Benchmark Protein Ladder (Invitrogen) and sizes of distinct bands are indicated. Lines across the gel indicate where the gel was severed in order to digest similar-sized proteins from both trophosomes.
(TIF)

Figure S3 **Ratio of first two peptide-spectral counts vs. ratio of second two peptide-spectral counts.** To calculate the averages, the number of peptides counted for a specific protein identification from East Pacific Rise (EPR) *Riftia pachpytila* were divided by the number of peptides from the Guaymas *Riftia pachpytila*. The majority of the data fall along a 1∶1 line, within the confidence intervals, suggesting that quadruplicate samples yielded comparable values and thus validating the usage of spectral counting for quantification.
(TIFF)

Table S1 **A subset of key metabolic proteins and their relative abundances in Guaymas and East Pacific Rise (EPR)** ***Riftia pachyptila*** **.** Peptide abundances are presented as the log_2_ of the normalized peptide counts for each proteome. Proteins more abundant in the Guaymas proteome are shaded pink. Those more abundant in the EPR proteome are shaded green.
(TIFF)
