# Supplementary figures and images for: Linking Hydrothermal Geochemistry to Organismal Physiology: Physiological Versatility in Riftia pachyptila from Sedimented and Basalt-hosted Vents
Source: PLoS One. 2011 Jul 14;6(7):e21692. doi: 10.1371/journal.pone.0021692 (PMC3136470; doi:10.1371/journal.pone.0021692)

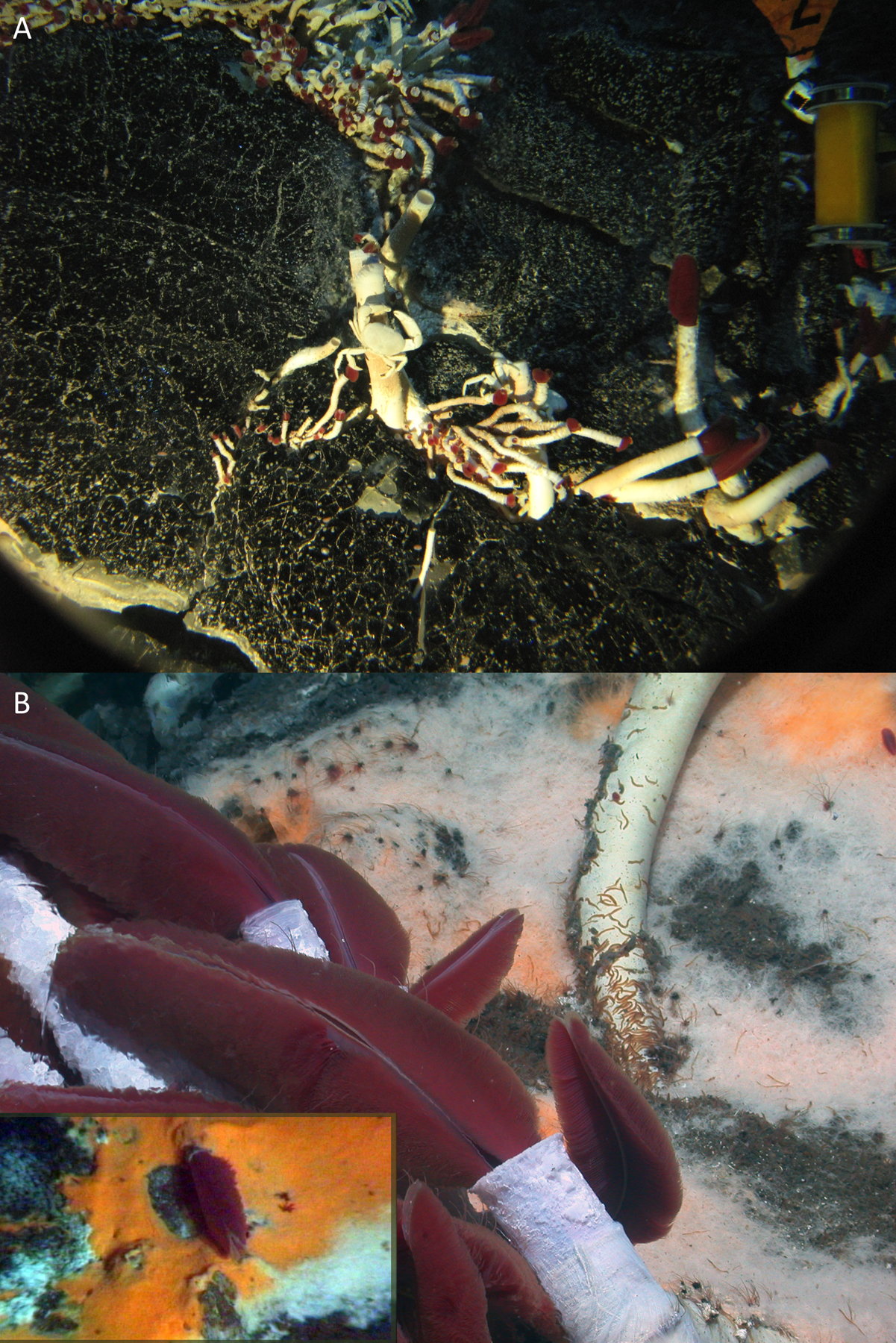

Supplement: Figure S1 — Riftia tubeworms observed at the East Pacific Rise (EPR) and the Guaymas basin. A) Riftia pachyptila observed on the basalt hosted vents at the EPR, B) Riftia pachyptila as observed at the sediment hosted vents in the Guaymas basin, 1b) inset show tubeworm buried in sediment to its plume. Images were taken during an expedition to the EPR and Guaymas in 2007. Figure 1b is from an expedition to the Guaymas basin in 2003 (courtesy of MBARI). (TIF) [file pone.0021692.s001.tif]

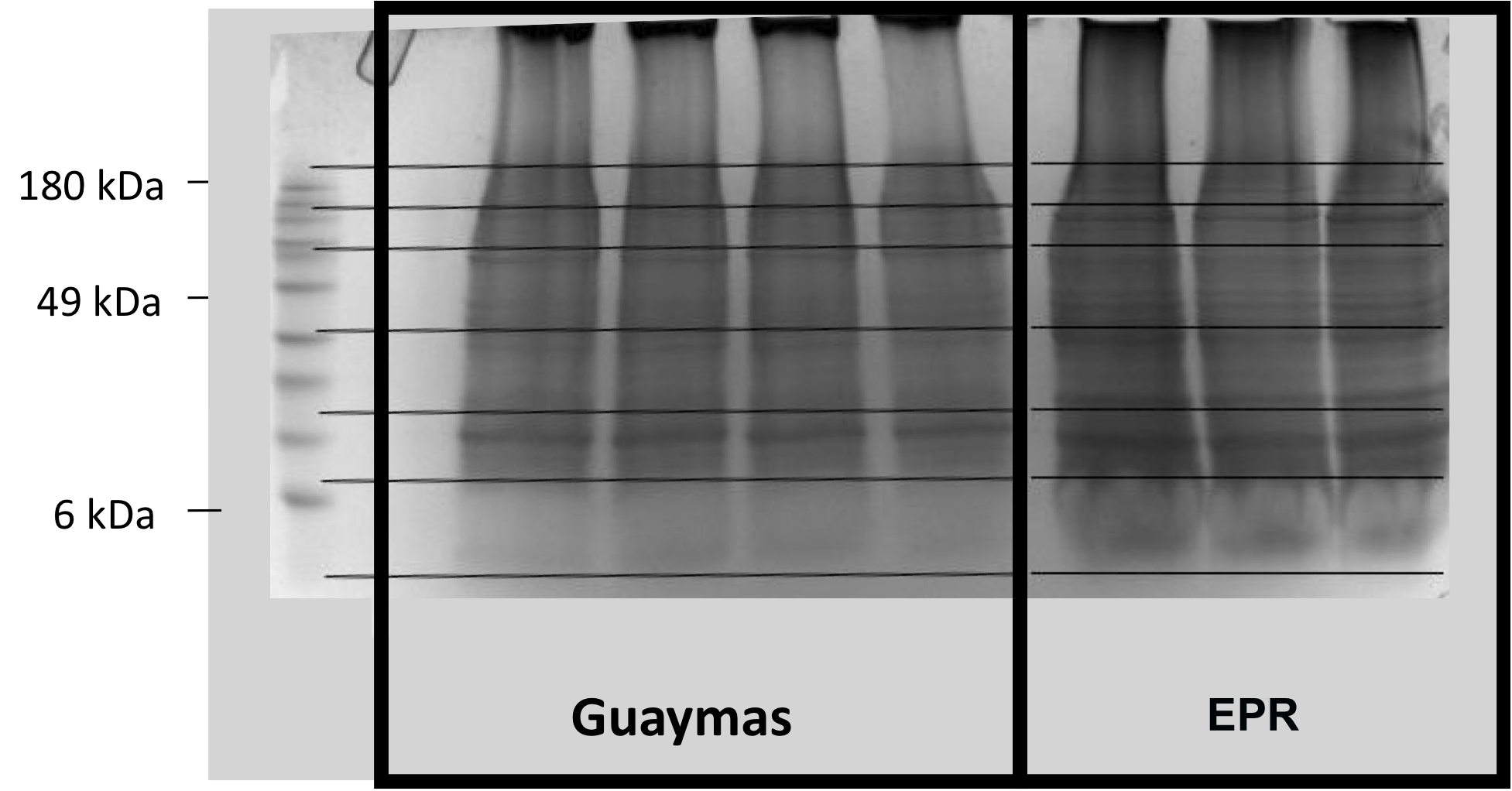

Supplement: Figure S2 — 15% SDS-PAGE gel of proteins recovered from Guaymas and East Pacific Rise (EPR) Riftia pachpytila trophosome. Marker shown is Benchmark Protein Ladder (Invitrogen) and sizes of distinct bands are indicated. Lines across the gel indicate where the gel was severed in order to digest similar-sized proteins from both trophosomes. (TIF) [file pone.0021692.s002.tif]

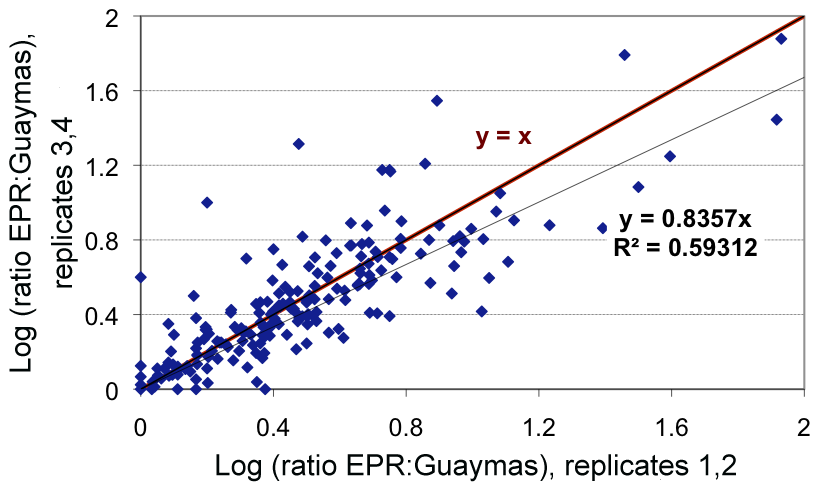

Supplement: Figure S3 — Ratio of first two peptide-spectral counts vs. ratio of second two peptide-spectral counts. To calculate the averages, the number of peptides counted for a specific protein identification from East Pacific Rise (EPR) Riftia pachpytila were divided by the number of peptides from the Guaymas Riftia pachpytila. The majority of the data fall along a 1∶1 line, within the confidence intervals, suggesting that quadruplicate samples yielded comparable values and thus validating the usage of spectral counting for quantification. (TIFF) [file pone.0021692.s003.tiff]

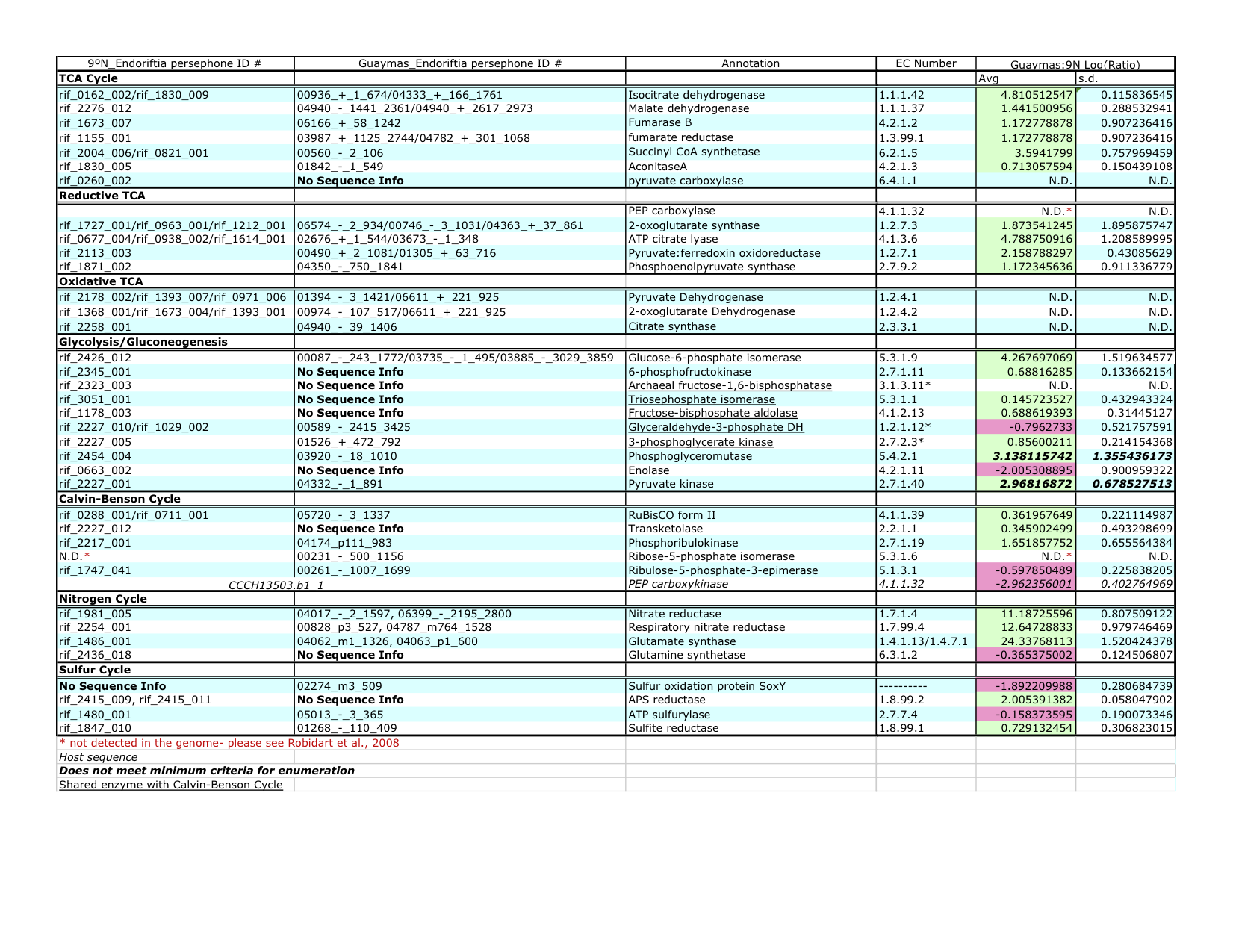

Supplement: Table S1 — A subset of key metabolic proteins and their relative abundances in Guaymas and East Pacific Rise (EPR) Riftia pachyptila . Peptide abundances are presented as the log2 of the normalized peptide counts for each proteome. Proteins more abundant in the Guaymas proteome are shaded pink. Those more abundant in the EPR proteome are shaded green. (TIFF) [file pone.0021692.s004.tiff]
